# Reviewed article: Curado DSP, Silva EV. Drugs incorporated into the Brazilian Unified Health System: documentary analysis of methodological decisions in budget impact analyses between 2012 and 2024. Epidemiol Serv Saude. 2025:34;e20250122.

**DOI:** 10.1590/S2237-96222025v34e20250122.c

**Published:** 2025-09-08

**Authors:** Ronaldo Portela

**Affiliations:** UFMG, Faculdade de Farmácia

**Table:** 

Rating	Excellent	Good	Average	Below average	Poor
Interest	✓				
Quality		✓			
Originality	✓				
Overall		✓			

**Table:** 

Questionnaire	Yes	No	Not applicable
Does the manuscript contain new and significant information to justify publication?	✓		
Does the Abstract (Summary) clearly and accurately describe the content of the article?	✓		
Is the problem significant and concisely stated?	✓		
Are the methods described comprehensively?	✓		
Are the interpretations and conclusions justified by the results?	✓		
Is adequate reference made to other work in the field?	✓		

## Manuscript Structure

Length of article is: Adequate

Number of tables is: Adequate

Number of figures is: Adequate

Please state any conflict(s) of interest that you have in relation to the review of this paper (state “none” if this is not applicable): None

## Comments

First of all, we are really grateful for the opportunity of this review.

This study is relevant because the analyses of the Budget Impact Assessments (AIO) allow the identification of possible improvements in CONITEC’s evaluation methodologies and processes. We now make our considerations: 

Page 3

Line 21 to 26 - Wasn’t there concern about the reports on private sector demands issued before 2019? Since there were no dossiers to be consulted.

Line 48 - To correct the sentence: Isto se deve ao fato dessas pessoas ainda…

Line 54 to 55 - To correct the sentence: As doenças oncológicas, por sua vez, não só estão acompanhadas de custos consideráveis, mas também de uma política de…

Page 4

Line 8 – The correct one, wouldn’t it be: … sendo superior à epidemiológica.

Page 5

Line 27 to 47 - We suggest the representation of the absolute values {X(%)} also in Figure 1 to facilitate the reading of the results.

Page 6

The possibility of biases related to the absence of dossiers referring to reports on private sector demands before to 2019 should be mentioned in the study’s discussion.

